# A comparison of isolated mandibular fractures and pattern of fractures of the mandible in cases with panfacial fractures – A study from 2007 to 2024 in a highest-level German trauma centre on 2056 patients with mandibular fractures

**DOI:** 10.1007/s10006-025-01478-5

**Published:** 2025-10-27

**Authors:** Lars Bonitz, Leonie Koch, Stefan Hassfeld, Ákos Bicsák

**Affiliations:** 1 University of Witten/Herdecke, Alfred-Herrhausen-Strasse 45, D-58453 Witten, Germany; 2 Clinic for Cranial- and Maxillofacial Surgery, Regional Plastic Surgery, Dortmund General Hospital, Muensterstrasse 240, D-44145 Dortmund, Germany

**Keywords:** Head and neck injury, Facial bone fracture, Mandible, Fracture, Demographics

## Abstract

**Introduction:**

This study investigates mandibular fractures focusing on long-term trends and fracture patterns of mandibular fractures as isolated fractures and as part of panfacial fractures.

**Methods:**

In addition to descriptive demographics, the fracture site distribution was statistically compared with the Wilcoxon rank test and compared in groups with the χ2-test.

**Results:**

2,699 mandibular fracture cases were analyzed: 1847 patients were isolated, and 852 with panfacial fractures involving the mandible. The male-to-female ratio was 3:1. Males were significantly younger than females in isolated and panfacial fractures. The yearly patient numbers decreased in panfacial cases and remained approximately the same – with high volatility – in isolated cases. Isolated fractures peaked in young males (20–30 years), while older females showed a secondary peak in panfacial fractures. Fracture distribution differed significantly between groups. A heatmap presents the fracture pattern in a schematic mandible, presenting both patient groups parallelly for better comparison. It highlights the cranialisation of the distribution pattern in the panfacial injury group.

**Summary:**

The distribution of mandibular fractures is different in isolated and panfacial fracture cases. The findings emphasize the importance of tailored diagnostic and treatment strategies for high-risk groups, including older females and patients with complex craniofacial injuries.

## Introduction

Most maxillofacial trauma studies were performed before the initiation of electronic vehicles, rollers, and bicycles after 2015. These vehicles are getting more popular lately. Thus, they gain importance in road traffic accidents [1, 2]. 2020–2022 the COVID-19 pandemics has brought lifestyle changes with lockdowns, social distancing, etc [3]. As these changes affect the whole society, it is inevitable, to perform re-evaluations if maxillofacial trauma epidemiology has changed. If there are relevant changes in healthcare planning, healthcare providers must also adopt new strategies.

The maxillofacial region is divided into three major parts: the upper face, the midface, and the lower face [4–7]. Due to didactic reasons, we performed our analysis once for the upper and midface region and once for the mandible. Fractures of the mandible can be single-site fractures, double-site fractures, multiple-site fractures, and comminuted fractures [8–11]. Mandibular fractures can be isolated fractures, e.g., fracture sites are located just in the mandible, or they can be part of panfacial fractures. The aim of our study was to analyze the long-term tendencies of mandibular fractures over 18 years of time. The second aim was to compare mandibular fracture distribution in isolated fracture cases and as part of panfacial fractures if these patients need special diagnostic and treatment approaches.

## Materials and methods

This study (No. 152/2017) has been approved by the Ethics Commission of the University of Witten/Herdecke.

This study was conducted in accordance with the Helsinki Declaration, the laws and regulations of the European Union, the Federal Republic of Germany, the State of North Rhine-Westphalia, and the General Hospital Dortmund.

### Diagnostic and treatment procedures

The diagnostic and treatment procedures were performed according to international standards. The fractures were always stated radiologically with at least two X-rays from two different directions or with a CT scan in at least two planes [12–14].

Surgical treatment was performed using mandibular-maxillary fixation intra- and postoperatively for reposition and post-operative fixation; the fracture reduction was performed via standard miniplate osteosynthesis or, in special cases, osteosynthesis using specialized mandibular plates or reconstruction plates [15–17]. Condyle fractures were fixed through a transoral or extern approach as per the surgeon’s preference.

Postoperatively, antibiotic coverage was administered using 3000 mg of ampicillin-sulbactam three times a day or equivalent in case of allergies.

### Classification of fractures, databank, statistics

The Dortmund Maxillofacial Trauma Registry is a Redcap-based [18, 19] databank that serves to analyze our patient data. Actually, a total number of 14,500 + patients have been included from the period of 2007–2024. The database lock was performed on the 30th of September, 2024.

The fracture classification follows the widely accepted AO Classification supplemented with the “Fracture of the anterior wall of the maxillary sinus” [6, 7, 20]. Data regarding demographics, medical history, concomitant medication, fracture data, and data regarding conservative or operative treatment were collected for each case.

For study purposes, demographic data, group comparisons with χ2-test and Wilcoxon ranked test were used. The statistical analysis was performed with SPSS 29.0 (IBM^®^). The results are presented in text form, tables, diagrams, and a heatmap.

## Results

The Ethics Commission of the University of Witten/Herdecke has approved this study (No. 152/2017). It was conducted in accordance with the Helsinki Declaration, the laws and regulations of the European Union, the Federal Republic of Germany, the State North-Rhine-Westphalia, and the General Hospital Dortmund.

**Fig. 1:**
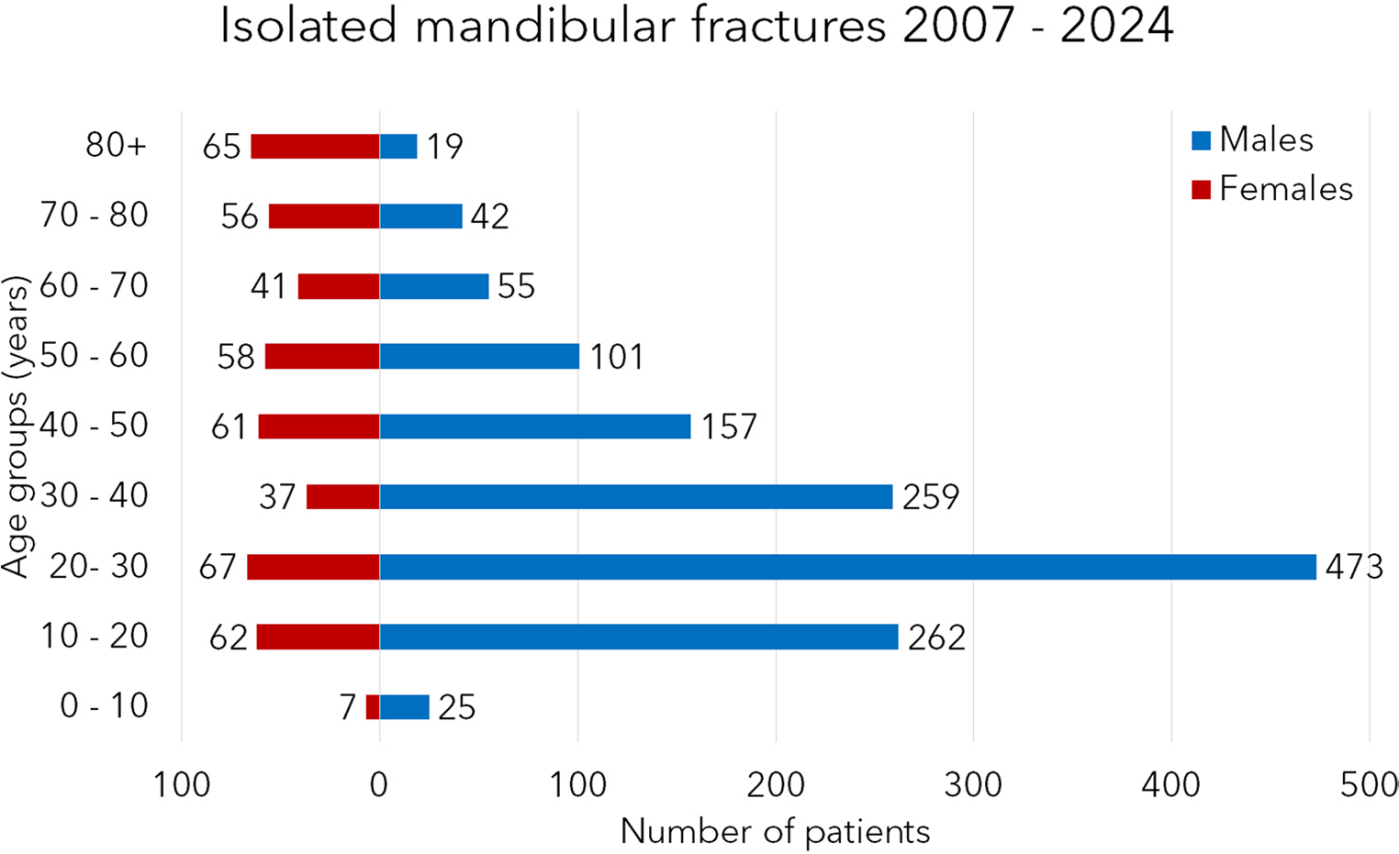
Histogram of the age related distribution of patients with isolated mandibular fractures

**Fig. 2:**
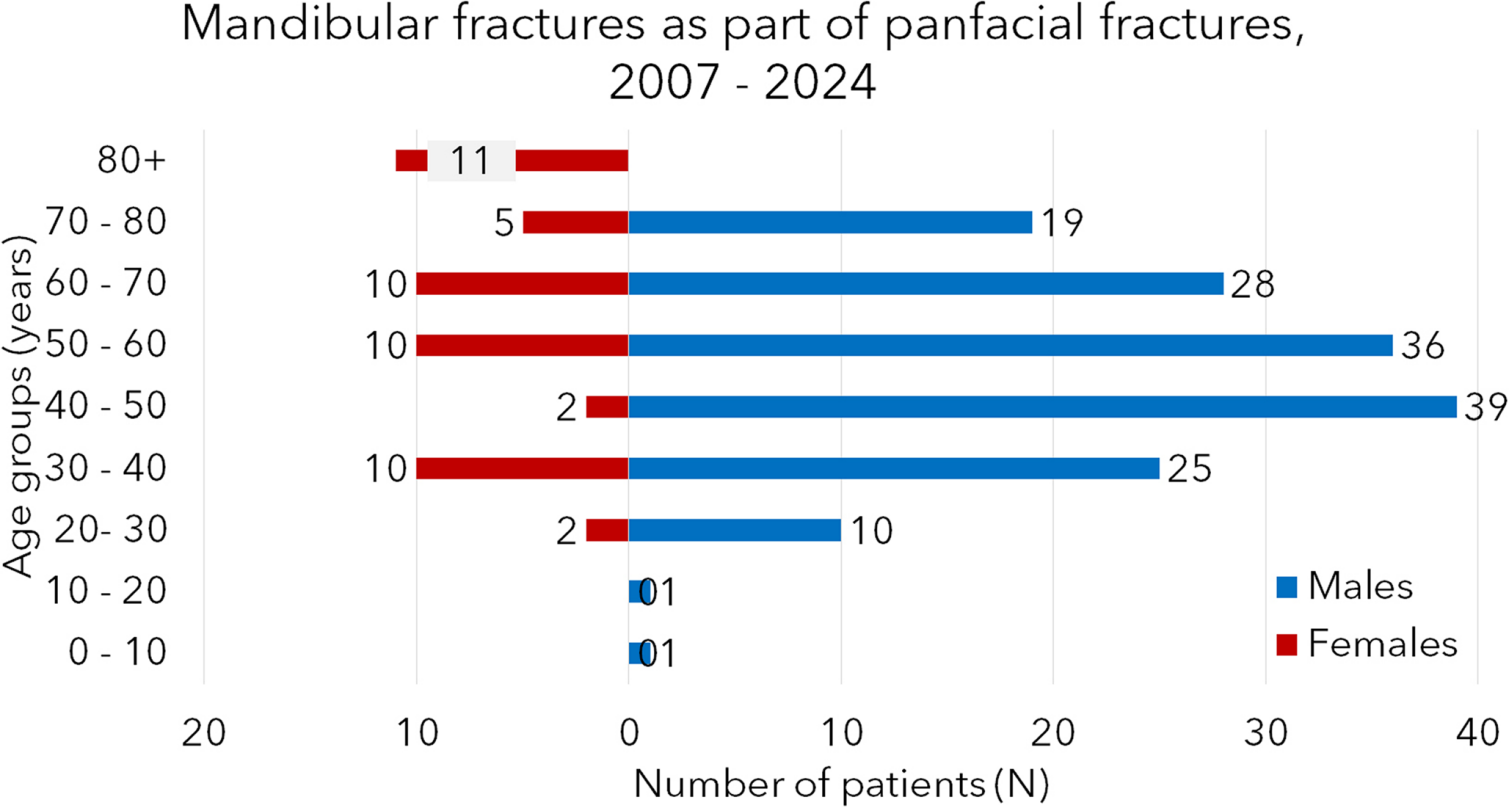
Age-related distribution of patients with mandibular fractures, with the mandibular fracture as part of panfacial fractures

The data was collected in the Dortmund Maxillofacial Trauma Registry, and the database lock took place on 30th September 2024. Table 1 shows the most critical demographic findings. In isolated fracture patients, 1393 males and 454 females were admitted, making a total of 1847 isolated cases and a male-to-female ratio of 3.1:1. In panfacial fractures, 154 males and 50 females were seen. This makes a total of 209 cases and a male-to-female ratio of 3.2:1. In the whole study, 1552 males and 504 females were included, which makes a total patient number of 2056 patients and a male-to-female ratio of 3.1:1. Male patients are younger in both patient groups (16 years younger in the isolated and 13 years younger in the panfacial group). Patients of the same gender with isolated fractures are younger than females (10 and 7 years, respectively). The histograms of Figs. 1 and 2 show the age-related distribution in both genders and patient groups. In females, young and old patients are most exposed to the fracture (especially well-seen in the panfacial histogram), while in males in isolated fractures from 20 to 30 y.o., in panfacial fractures, 40–50-year-old males are the most exposed to mandibular fractures. The difference in the number of patients between both genders was found statistically significant in both patient groups and age groups (χ^2^-test, *p* < 0.05 in all cases). Data from 2024 includes patients from the period of January to September only.

**Table 1:** The most important demographic data of patients with mandibular fracture(s) is presented

Gender	Isolated fractures		Panfacial fractures		Total	
	*N*	Age (average, SD, years)	*N*	Age (average, SD, years)	*N*	Age (average, SD, years)
Male	1393	33.1 ± 16.6	159	39.5 ± 17.9	1552	33.8 ± 16.7
Female	454	49.3 ± 24.3	50	52.2 ± 23.9	504	49.7 ± 24.2
*Total*	*1847*	*37.1 ± 20.0*	*209*	*42.5 ± 20.2*	*2056*	*37.7 ± 20.0*

“Isolated fractures” refer to patients with isolated mandibular fracture(s), and “Panfacial fractures” reflect patients with mandibular fractures *and* at least one more fracture in the midface and/or forehead region. If two or more mandibular fractures are present, these are always referred to in both patient groups as “multiple fractures”

**Fig. 3:**
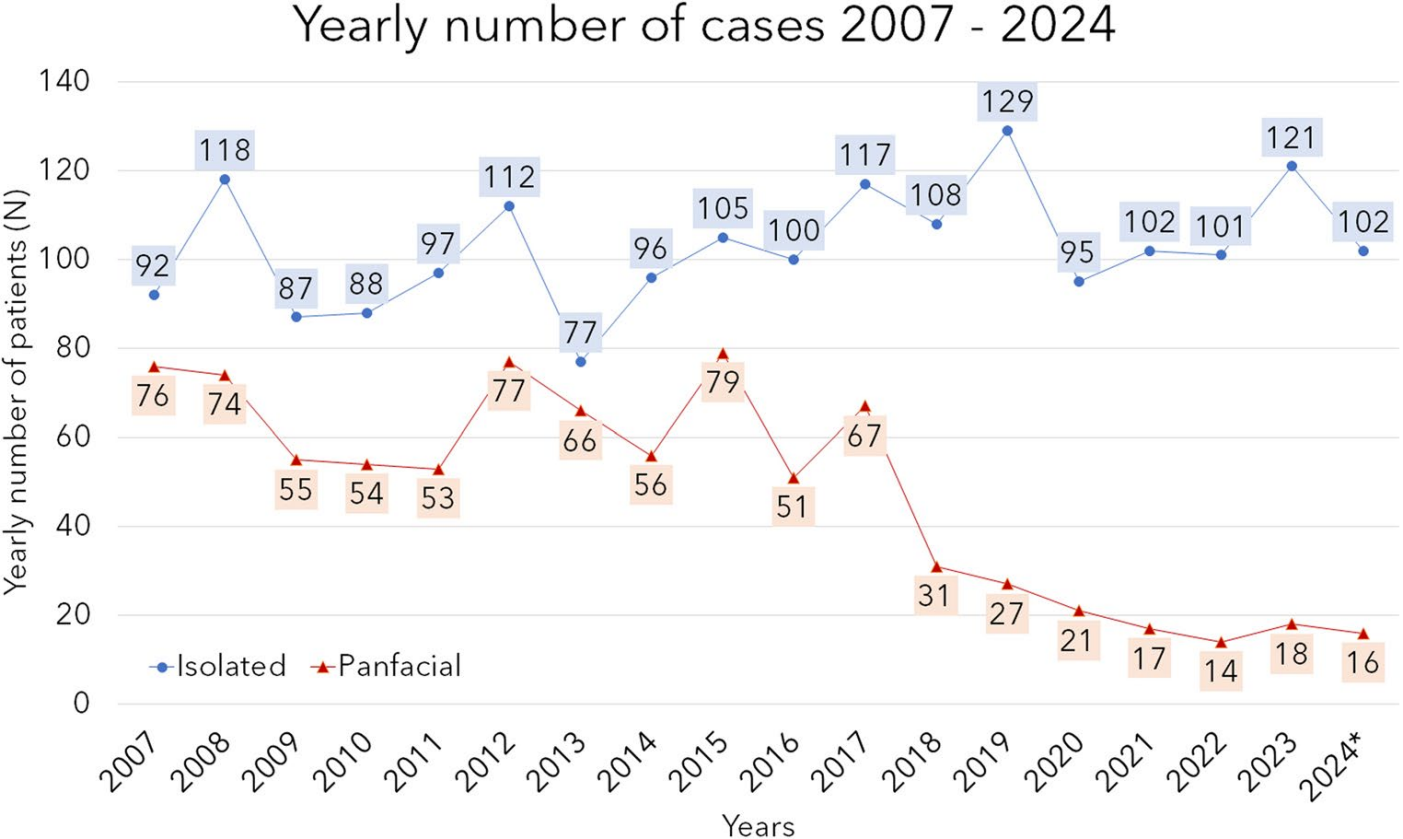
Yearly case numbers (number of patients treated for mandibular fractures as isolated fractures or as part of panfacial fractures, absolute numbers)

Figure 3 shows the yearly number of patients over the whole period (2024 data refers to January to September only). In this diagram, a significant difference between the two patient groups can be observed. After 2017, the number of panfacial cases showed a decreasing tendency. The curve of the isolated fractures shows a dip in 2020, the first year of the SARS-CoV-2 pandemic, and an increasing tendency with higher volatility since 2021.

**Fig. 4:**
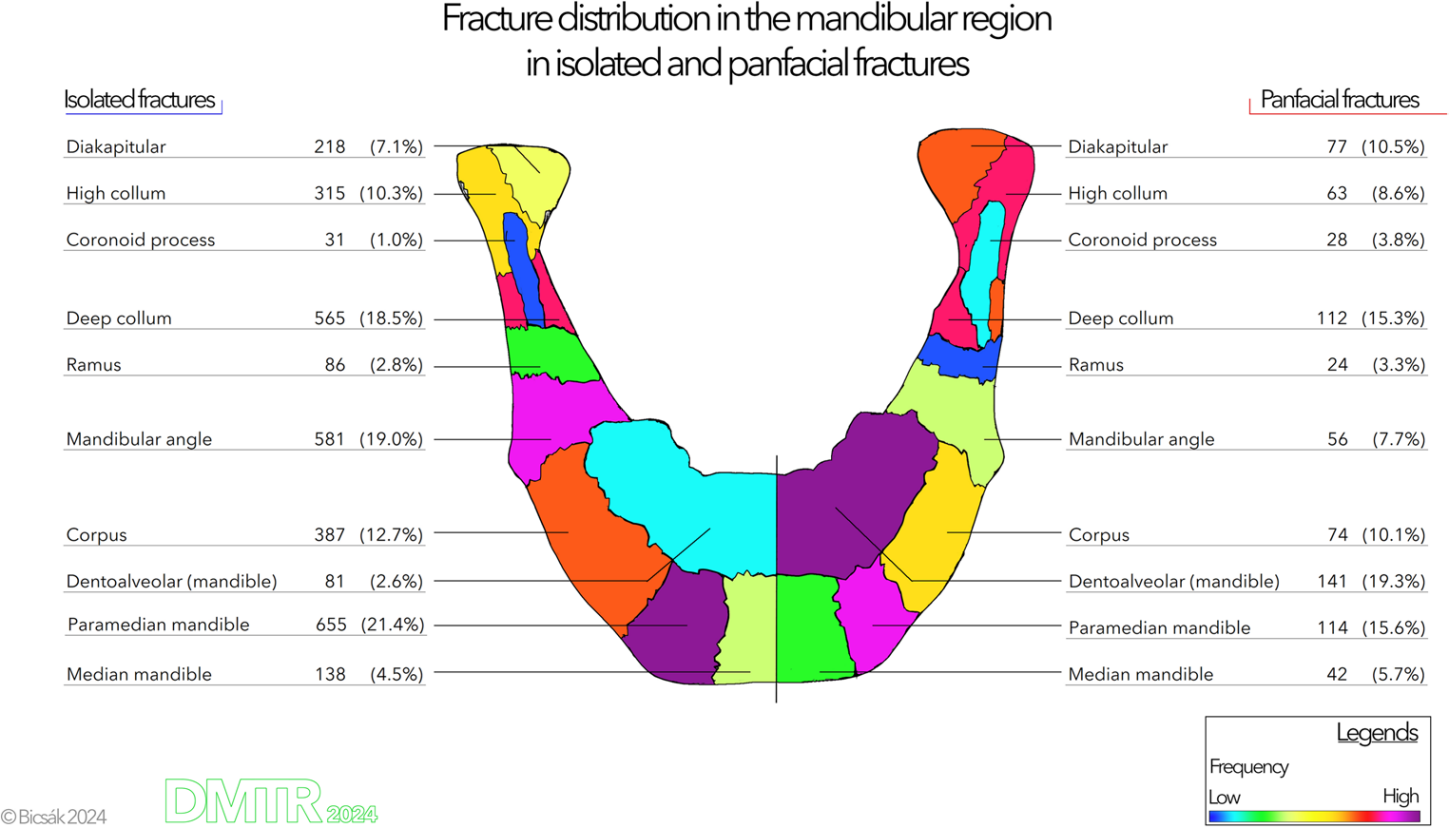
Fracture distribution heatmap of mandibular fractures. The color coding and % values refer to the total number of fractures in the same group (isolated fractures or as part of panfacial fractures). Please note that the left and right sides of the picture represent both patient groups and not the laterality of fractures (left and right fractures are summarized)

Figure 4 represents the distribution heatmap of mandibular fractures in both patient groups. In isolated fractures, most fractures are located in the paramedian area, mandibular angle, and lower collum area (respectively 21.4%, 19.0%, and 18.5% of the fractures). The right side of the figure shows the distribution matrix in panfacial cases. Here, most fractures occurred in the dentoalveolar area (19.3%), then in the paramedian area (15.6%), followed by the whole articular process. This, in summary, means a clear cranialization of the fractures. The statistical comparison of the two distributions with the Wilcoxon ranked test showed a highly significant difference (*p* = 0.005). 47% of the cases included one fracture site, 41% two fracture sites, and 10% three of them; in the rest of the cases, three, four, five, six, or eight fracture sites were found.

## Discussion

The Dortmund Maxillofacial Trauma Registry is one of the biggest monocentric databases worldwide, with over 14,500 individual patient datasets. The monocentric study has its advantages and disadvantages. A clear advantage is the homogeneity of our data, the same and consequent classification system, and the possibility of increasing data quality. The clear disadvantage is the missing comparability. The study’s retrospective nature is also a limiting factor in assessing trends. As trauma data cannot be presented prospectively, the study achieves the highest possible evidence level within its design limits. As our other study with the German Trauma Registry (DGU^®^) has shown, our centre data is statistically comparable with the data from Germany, Austria, and Switzerland and, therefore, represents an adequate sample of head and neck injuries. We assume that, in return, our data can also be extrapolated to the three above countries with an approximate inhabitant number of approximately 100 million [21].

The study timeline contains data before, during and after the COVID-19 pandemics. In our sample, the number of patients in the first pandemics year decreased 26% in comparison to 2019 and 20% in comparison to the average patient number in the years 2017–2019. This change fits to the literature, Alves et al. found 24% decrease in their review [22]. In international literature similar decrease was observed after implementing social distancing in 2020–2021. In the UK in this period, a clear change was found: the number of injured and the impact of the trauma decreased, the injury distribution has shown a different pattern [23]. Other demographic, epidemiologic or treatment factors were similar to those of our study [22–26]. There are much less data about post-pandemic epidemiology. We observed an increase in the number of patients nearly as high as in the pre-pandemic years. Similar is reported in literature [27].

The comparison of the two study groups showed significant differences. The isolated fracture group is in both genders younger than the panfacial group (6 and 3 years in males and in females respectively). Also, within study groups, males are much younger than females (16 and 13 years, respectively, in the isolated and panfacial groups). The male-to-female ratio is, however, very stable and comparable with literature data [8, 28–30].

The age group distribution is very similar in the two groups. The most exposed are young males; the peak is in the age group of 20–30-year-olds. In females, the peak is smaller, and there is a double peak in the same age and in the age group of 80-year-olds and older. This second peak is much more marked in the panfacial group. As our previous studies and international papers stated, older females are a high-risk fracture group and a high-risk group for concomitant injuries [31, 32].

The fracture distribution in the mandibular area was statistically different between the two patient groups. Also, the visualization in Fig. 4 shows a more cranial distribution of the fracture sites. This can result from the different biomechanics of panfacial fractures (higher forces, other directions etc.). Similar distribution with an increased number of fractures of the articular process (and settlement of the most fractures to the cranial base or Frankfurt horizontal) was found in cases with concomitant cervical spine injuries both in our study and in the international literature [31–35]. Here, we must point out again that patients with panfacial fractures and older females with head and neck injuries are high-risk patients for suffering a concomitant cervical spine injury. Therefore, we suggest performing an initial CT scan of the complete head, cervical spine, and facial region. The different fracture distribution pattern should be taken into consideration during the first clinical examination, especially, if the patient is unconscious. The presented heatmap can warn all physicians working in the emergency department to exclude further injuries. It is very important to recognize fractures of the mandible near to the cranial base and to detect concomitant injuries to the auditory canal, cranial base or injuries to other cervical structures.

The fracture distribution in isolated cases is similar to those in the international literature [8, 29, 36–40]. In our study, 47% of the cases showed one fracture site, and in 53%, two or more, up to eight fractures. This means that if a fracture of the mandible was found, a second fracture should be excluded to be able to state the single fracture site.

## Conclusion

This study has shown a slightly increasing tendency in isolated mandibular fractures during the last years and a decreasing number of panfacial fracture cases. The results showed the importance of the biomechanics of fractures. Together with our paper on midface and forehead fractures, the significant difference in the fracture distribution and its re-distribution to the cranial base area may indicate an important biomechanical role of the cranial base, especially in the distribution and conduction of forces and load during trauma. Further studies are required if there is a possibility to develop new safety devices for road traffic participants or patients with susceptibility to falls.

## Data Availability

No datasets were generated or analysed during the current study.
